# MPF Governs the Assembly and Contraction of Actomyosin Rings by Activating RhoA and MAPK during Chemical-Induced Cytokinesis of Goat Oocytes

**DOI:** 10.1371/journal.pone.0012706

**Published:** 2010-09-13

**Authors:** Yan-Guang Wu, Ping Zhou, Guo-Cheng Lan, Da Gao, Qing Li, De-Li Wei, Hui-Li Wang, Jing-He Tan

**Affiliations:** College of Animal Science and Veterinary Medicine, Shandong Agricultural University, Tai-an City, People's Republic of China; University of Birmingham, United Kingdom

## Abstract

The interplay between maturation-promoting factor (MPF), mitogen-activated protein kinase (MAPK) and Rho GTPase during actin-myosin interactions has yet to be determined. The mechanism by which microtubule disrupters induce the formation of ooplasmic protrusion during chemical-assisted enucleation of mammalian oocytes is unknown. Moreover, a suitable model is urgently needed for the study of cytokinesis. We have established a model of chemical-induced cytokinesis and have studied the signaling events leading to cytokinesis using this model. The results suggested that microtubule inhibitors activated MPF, which induced actomyosin assembly (formation of ooplasmic protrusion) by activating RhoA and thus MAPK. While MAPK controlled actin recruitment on its own, MPF promoted myosin enrichment by activating RhoA and MAPK. A further chemical treatment of oocytes with protrusions induced constriction of the actomyosin ring by inactivating MPF while activating RhoA. In conclusion, the present data suggested that the assembly and contraction of the actomyosin ring were two separable steps: while an increase in MPF activity promoted the assembly through RhoA-mediated activation of MAPK, a decrease in MPF activity triggered contraction of the ring by activating RhoA.

## Introduction

Cytokinesis is mediated by a dynamic interplay between the microtubules of the mitotic spindle, the actomyosin cytoskeleton and membrane fusion events [1]. For many decades, although morphological observations led to great insights into the cellular structures that orchestrate cell division, the underlying molecular machinery is largely unknown. While studies suggest that a local minimum of microtubule density or microtubule depolymerization induces the formation of contractile rings through activation of RhoA [2], [3], how RhoA is activated has yet to be determined. In addition, although both the maturation-promoting factor (MPF) and mitogen-activated protein kinase (MAPK) were found to regulate actin-myosin interactions [4]–[6], interplay between these two kinases in this context has not been reported. Furthermore, unlike other cell cycle events, cytokinesis has been particularly resistant to in vitro biochemical approaches, making research progress very slow [7]. A powerful in vitro model is therefore urgently needed to dissect out the different steps and molecules involved in cytokinesis.

During meiotic maturation of mammalian oocytes, the meiosis I spindle first assembles around the centrally positioned chromosomes and then migrates to the cortex of the oocyte [8], [9]. Meanwhile, an actin-rich but cortical granule- and microvillus-free cortical domain develops over the eccentrically positioned meiotic spindle [10]–[12]. While in most mitotic cells, the cues that direct cell polarization are often extrinsic, coming from the environment or certain cortical landmarks [13], [14], the molecular cues for asymmetric meiotic divisions of oocytes remain poorly understood. Although studies suggested that the subcortically positioned chromosomes in mouse oocytes [12], [15], [16], or the attachment of spindle pole to the cortex of Xenopus oocytes [17], [18], could provide the necessary cue, and that the small GTPases [16]–[19] might be involved, it is unknown how chromosomes or the spindle pole attachment activate small GTPases during the establishment of cortical polarity and assembly of a contractile ring.

A brief treatment of matured oocytes with microtubule disrupter demecolcine results in a cytoplasmic protrusion containing a condensed chromosome mass [20]–[22]. In goat oocytes with demecolcine-induced ooplasmic protrusions the spindle disintegrated and a contractile ring formed around the condensed chromosome mass [22]. When these oocytes were treated with cytochalasin B, the contractile ring disappeared while the spindle reintegrated. Therefore, we proposed that if the chemical-induced ooplasmic protrusion can pinch off after a further appropriate treatment, the goat oocyte could serve as an in vitro model for study of cytokinesis. We have established such a model and have studied the interactions between MPF, RhoA and MAPK on the assembly (ooplasmic protrusion) and contraction (extrusion of second polar bodies, Pb2) of the actomyosin ring using this model. The results suggested that the assembly and contraction of the ring were two separable steps: while an increase in MPF activities promoted the assembly through RhoA-mediated activation of MAPK, a decrease in MPF activity induced the contraction of the ring also by activating RhoA.

## Results

### Demecolcine-induced ooplasmic protrusion was associated with activation of both MPF and MAPK

**Figure 1:**
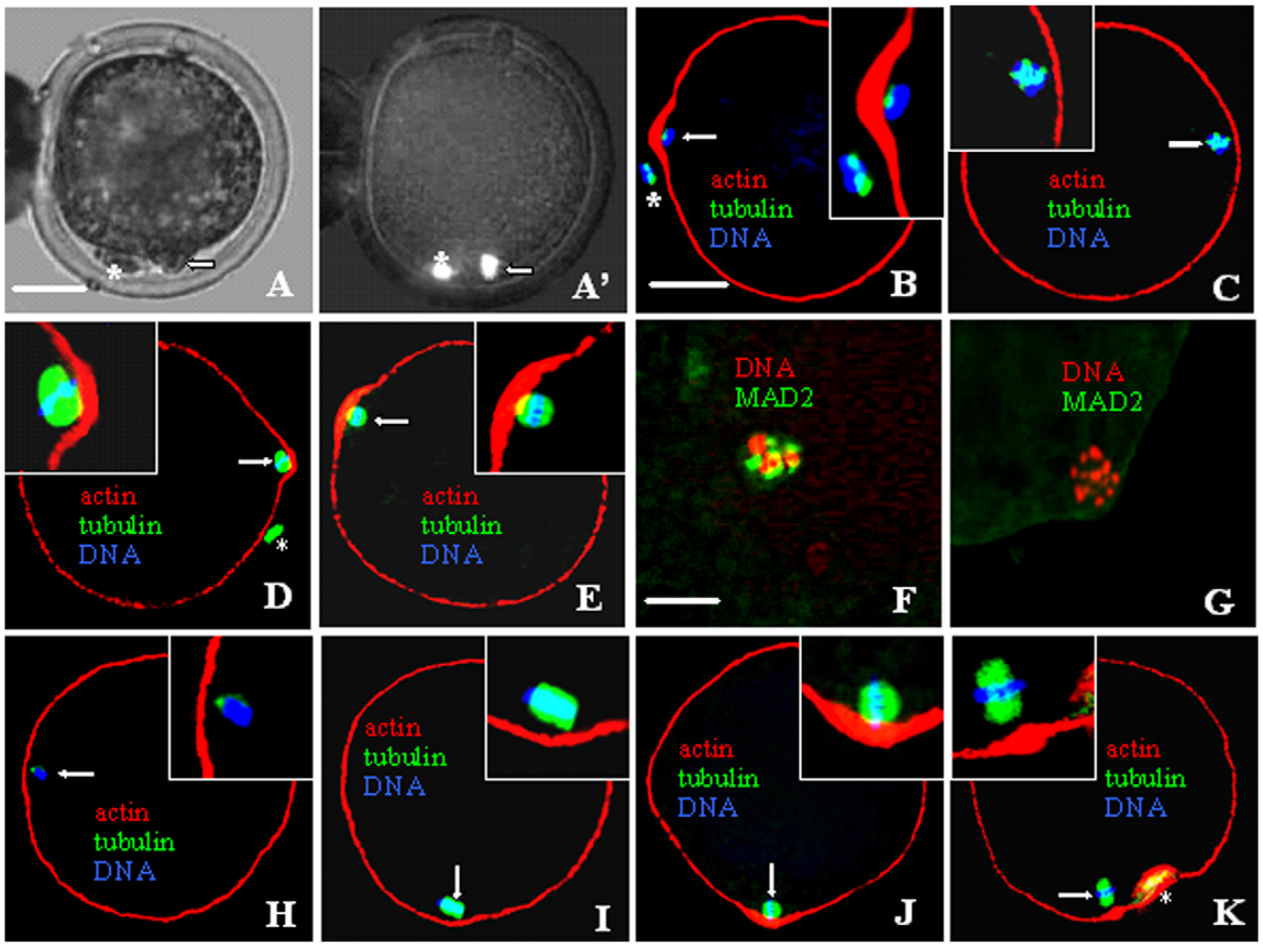
Photographs of goat oocytes after different treatments to induce ooplasmic protrusions. A and A' are the same oocyte observed under phase contrast and fluorescence microscope, respectively, after Hoechst staining. B to K are confocal micrographs, of which, B to E and H to K are merged images with DNA colored blue, α-tubulin green and actin red while F and G with DNA colored red while the activated MAD2 green. A and B show oocytes with protrusions while C shows an oocyte without protrusion after demecolcine treatment. D and E are oocytes with protrusions after treatment with MG132 and caffeine, respectively. F and G are oocytes MAD2-positive and -negative after demecolcine and MG132 treatment, respectively. H and I show D+U- and M+U-treated oocytes with spindles disassembled and intact, respectively. J and K are OA.5h oocytes without protrusion and OA4h oocytes with protrusion, respectively, both showing actin enrichment. Arrow: intact or disintegrated spindles; *: Pb1. The scale bar is 30-µm in A–E and H–K but 70-µm in F and G.

**Figure 2:**
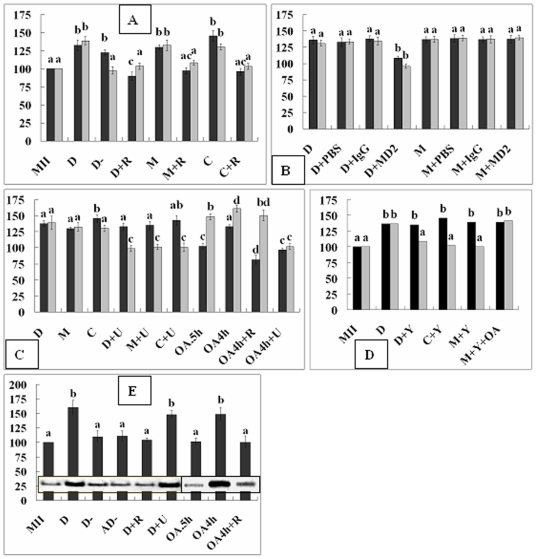
Relative MPF and MAPK activities (A to D) and RhoA-GTP levels (E) in oocytes after different treatments. In figures A to D, black and gray bars represent MPF and MAPK activities, respectively. MII: Freshly matured oocytes; D and D-: Oocytes with and without protrusion respectively after demecolcine; AD-: Aged oocytes without protrusion after demecolcine; +: In the presence of; R: ROS; U: U0126; Y: Y27632; OA.5h and OA4h: OA for 0.5 and 4 h respectively; MD2: MAD2. a–d: Values without a common letter above their bars differ (P<0.05) within enzyme activities.

After a 30-min treatment with 0.8 ng/ml demecolcine, 85% of the goat oocytes showed ooplasmic protrusions with a condensed chromosome mass (Figure 1A and A'). While all the oocytes with protrusions (n = 40) showed a disintegrated spindle and an actin-enriched ring around the condensed chromosome mass (Figure 1B), spindles were disintegrated but no actin ring was observed in oocytes without protrusions (Figure 1C). Compared to that in control oocytes, while both MPF and MAPK activities increased significantly in oocytes with protrusions, only the MPF activity increased in oocytes without protrusions after demecolcine treatment (Figure 2A).

### Demecolcine induced ooplasmic protrusion by increasing MPF and MAPK activities by activating MAD2

While over 80% of the oocytes treated with demecolcine, MG132 (5-µM) or caffeine (1-mM) formed protrusions in the absence of ROS, less than 5% did in the presence of 400-µM ROS. While both MPF and MAPK activities increased significantly in oocytes with protrusions, neither increased when protrusion was inhibited by ROS (Figure 2A). In contrast to those in demecolcine-treated oocytes, spindles in the MG132- or caffeine-treated oocytes were not disintegrated (Figure 1D and E). While 95% of the demecolcine-treated oocytes were MAD2-postive (Figure 1F), all the oocytes with MG132- or caffeine-induced protrusions were MAD2-negative (Figure 1G). Injection of anti-MAD2 antibodies decreased protrusion (23%) and inhibited activation of both MPF and MAPK (Figure 2B) in the demecolcine-treated, but not in MG132-treated oocytes.

### MPF induced ooplasmic protrusion by increasing MAPK activities

When oocytes were treated with demecolcine, MG132 or caffeine in the presence of 20-µM U0126, protrusion was inhibited (<7%); while MPF activities increased, the MAPK activity did not (Figure 2C). Spindles were disassembled in demecolcine+U0126 treated oocytes (Figure 1H) but intact in the MG132+U0126 treated oocytes (Figure 1I). While only 23% of the oocytes formed protrusions when treated with 0.5-µM OA for 0.5 h (OA.5h), 78% did when treated with 0.1-µM OA for 4 h (OA4h). In oocytes without protrusions after OA.5h treatment, while the MPF activity did not change, MAPK activities increased significantly (Figure 2C). In oocytes with protrusions after an OA4h treatment, however, both kinase activities increased. The OA.5h oocytes without protrusions showed actin enrichment and intact spindles (Figure 1J). Although both ROS and U0126 inhibited protrusion in the OA4h oocytes (<6%), ROS prevented activation of only the MPF activity while U0126 inhibited both MAPK and MPF (Figure 2C). The results suggested that (1) MPF induced ooplasmic protrusion by increasing MAPK activities and (2) OA activated MPF by way of MAPK.

### MPF activates MAPK by way of RhoA during ooplasmic protrusion

When oocytes were treated with demecolcine, MG132 or caffeine in the presence of 400-µM Y27632 (inhibitor for Rho-kinase ROCK), protrusion was inhibited (10%), and while MPF activities increased, the MAPK activity was unchanged (Figure 2D). Treatments that increased both MPF and MAPK activities (D and OA4h) activated RhoA (Figure 2E); treatments that increased MPF but not MAPK activities did not activate RhoA (D- and AD-); and treatment that did not increase MPF activities did not activate RhoA whether it increased MAPK activities (OA.5h and OA4h+R) or not (D+R). RhoA was activated in oocytes treated with demecolcine and U0126 (D+U) though their MAPK activation was inhibited. This suggested that (1) MPF activated MAPK by way of RhoA during ooplasmic protrusion and (2) OA activated while U0126 inactivated MAPK downstream of RhoA.

### Interactions between MPF, MAPK and RhoA during the assembly of actomyosin rings

**Figure 3:**
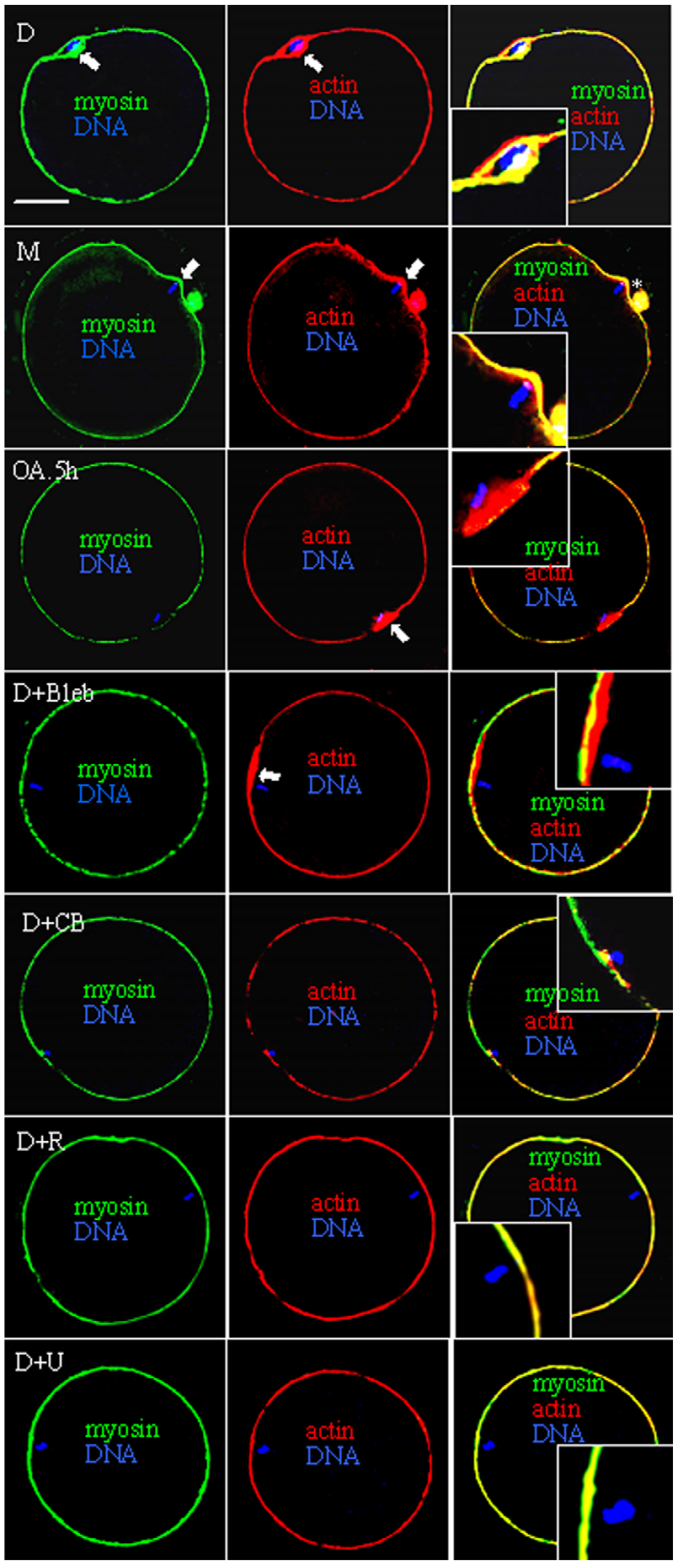
Confocal micrographs of oocytes showing enrichment of actin and/or myosin (arrow) after different treatments. Myosin is shown in green, actin in red and DNA in blue. Treatments include demecolcine (D), MG132 (M) or OA for 0.5 h (OA.5h) alone or demecolcine + Blebbistatin (Bleb), Cytochalasin B (CB), U0126 (U) or ROS (R). *: Pb1. Scale bar is 30 µm.

Treatment with 40-µg/ml CB (actin inhibitor) or 200-µM blebbistatin (myosin II inhibitor) completely inhibited demecolcine- or MG132-induced ooplasmic protrusion. While oocytes with protrusions always showed enrichment of both actin and myosin in the protruding domain (Figure 3), oocytes without protrusions showed either only actin enrichment (D+Bleb) or neither actin- nor myosin-enrichment (D+CB). Treatments that inhibited both MPF and MAPK (D+R) or MAPK alone (D+U) prevented enrichment of both myosin and actin, whereas treatment that inhibited MPF while activating MAPK (OA.5h) allowed actin but not myosin enrichment. Neither actin nor myosin was enriched in oocytes without protrusions after demecolcine+Y27632 treatment (data not shown). When oocytes were treated with MG132 in the presence of OA and Y27632 (M+Y+OA), both MPF and MAPK activities increased (Figure 2D), but protrusion was inhibited (7%) and only actin enriched while myosin did not (data not shown). The results suggested that (1) OA activated MAPK downstream of ROCK; (2) while MAPK promoted actin enrichment on its own, MPF promoted myosin enrichment in a ROCK- and MAPK-dependent manner.

### Chromatin is essential for chemical induction of actomyosin assembly

**Figure 4:**
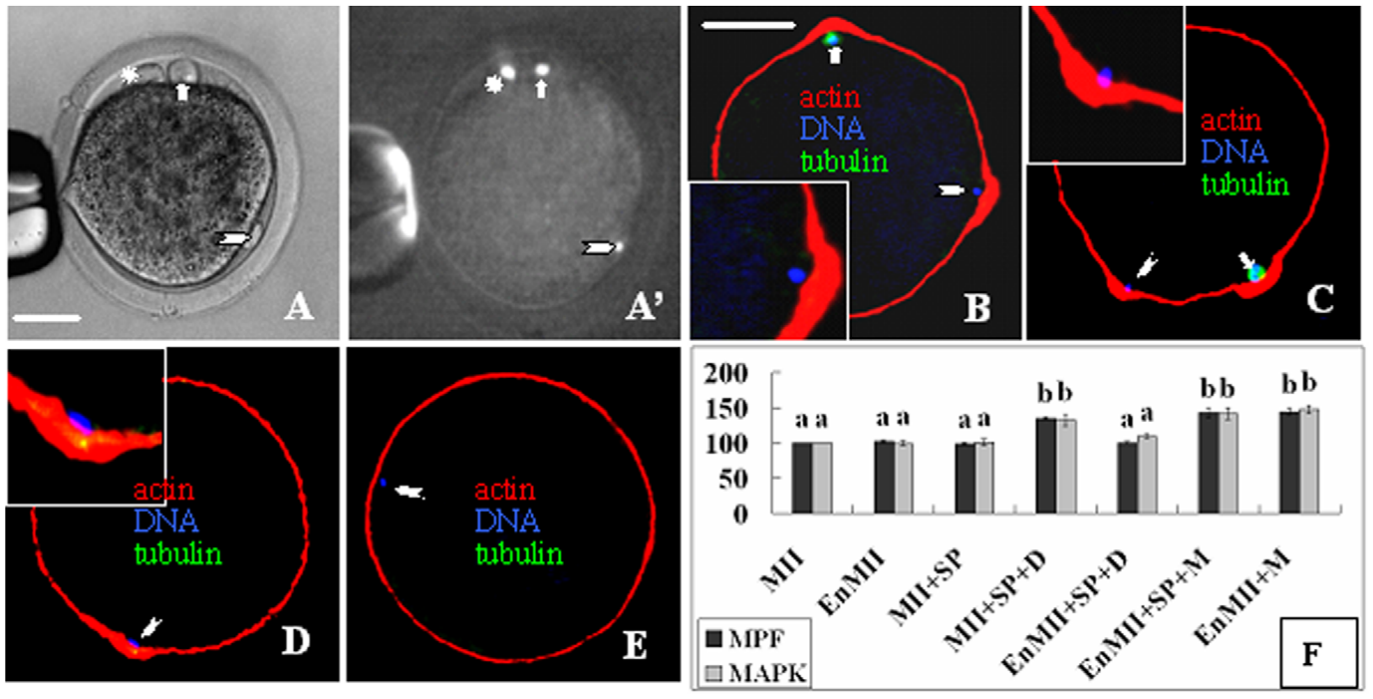
Ooplasmic protrusion and MPF/MAPK activities after oocytes were injected with mouse sperm head (SP) and treated with demecolcine or MG132. A and A' are the same oocyte observed under phase contrast and fluorescent microscopes, respectively, showing the egg chromosome-induced protrusion (arrow), SP-induced protrusion (arrowhead) and Pb1 (*). B to E show confocal images with DNA colored blue, α-tubulin green and actin red. B and C: Oocytes with both egg chromosome- and SP-induced protrusions after demecolcine and MG132 treatment, respectively. D and E: Enucleated oocytes injected with SP formed protrusion after MG132 (D) but did not after demecolcine (E). Scale bar is 20 µm. F: Relative MPF/MAPK activities after intact (MII) or enucleated oocytes (EnMII) were injected with SP (+SP) and treated with demecolcine (+SP+D) or MG132 (+M). Values without a common letter above their bars differ (P<0.05) within kinase activities.

While injection of mouse sperm tails had no effect, injection of mouse sperm head into intact goat oocytes induced a second ooplasmic protrusion (Figure 4A and A') in about half of the injected oocytes after treatment with demecolcine (Figure 4B) or MG132 (Figure 4C). The oocyte spindle is disintegrated after demecolcine but intact after MG132 treatment. When mouse sperm heads were injected into enucleated oocytes, however, protrusion was observed only after MG132 (Figure 4D) but not demecolcine treatment (Figure 4E).The MPF and MAPK activities increased significantly in intact but not in enucleated oocytes after sperm head injection and demecolcine treatment (Figure 4F). No protrusion formed when enucleated oocytes were treated with MG132 although both MPF and MAPK activities increased significantly. The results suggested that ooplasmic protrusion required interactions between chromatin and the signaling events and the role of chromatin in inducing actomyosin assembly was non-species-specific.

### Interactions between MPF, MAPK and RhoA during the constriction of actomyosin rings

**Figure 5:**
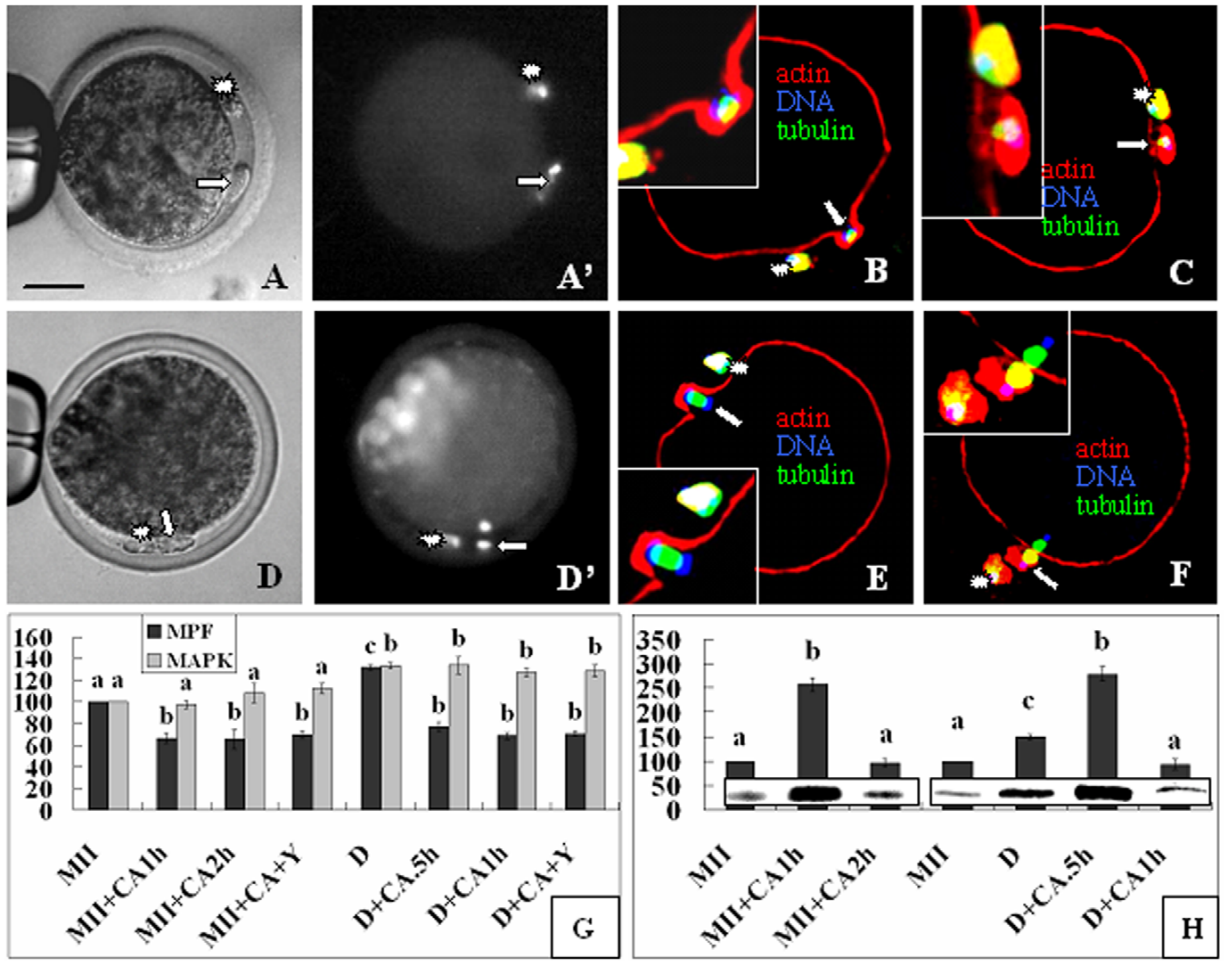
Pb2 Extrusion and activities of MPF, MAPK and RhoA after oocytes with demecolcine-induced protrusions (DIP) were activated. A, A' and D, D' are the same oocyte observed under phase contrast and fluorescence microscope, respectively, after Hoechst staining. B, C, E and F are confocal images with DNA colored blue, α-tubulin green and actin red. Contraction of the actomyosin ring began at 0.5 h (B) and 1 h (E) while Pb2 extrusion (arrows) observed at 1 h (A, A', C) and 2 h (D, D', F) after chemical activation (CA) of the DIP and control MII oocytes, respectively. *: Pb1. Scale bar is 30 µm. Panels G and H show relative MPF/MAPK and RhoA activities, respectively. MII and D: Freshly matured and DIP oocytes prior to CA; +: In the presence of; Y: Y27632; CA.5h, CA1h and CA2h: Oocytes assayed at 0.5, 1 and 2 h of CA, respectively. a–d: Values without a common letter above their bars differ (P<0.05) within enzyme activities.

Contraction of the actomyosin ring (Figure 5B and E) began at 0.5 and 1 h while extrusion of Pb2 (Figure 5A and D) was observed at 1 and 2 h after chemical activation of oocytes with demecolcine-induced protrusions and the control MII oocytes, respectively. While the Pb2 in control oocytes were extruded with half of the chromosome complement with the other half left inside the oocyte (Figure 5D' and F), the demecolcine-induced protrusions were pinched off with the whole complement of chromosomes (Figure 5A' and C). While 63% of the demecolcine-treated and 87% of the control oocytes released Pb2 after chemical activation in the absence of Y27632, the percentage decreased to 4% and 12%, respectively, in the presence of Y27632. While MPF activities declined to the lowest level at the time when the ring began to contract, the MAPK activity remained high until the time for Pb2 extrusion in both demecolcine-treated and control oocytes (Figure 5G). Y27632 affected neither the MPF nor MAPK dynamics. RhoA-GTP increased to the highest level at the time for ring contraction but decreased to the basal level by the time for Pb2 extrusion in both oocyte groups (Figure 5H).

## Discussion

In this study, both MPF and MAPK activities increased significantly in oocytes with protrusions after demecolcine treatment. While injection of mouse sperm head into intact goat oocytes induced a second ooplasmic protrusion and a significant increase in both MPF and MAPK activities, injection into enucleated oocytes had no effect after demecolcine treatment. Furthermore, while MG132 or caffeine induced ooplasmic protrusion without destroying spindles, treatment with ROS inhibited protrusion in oocytes with demecolcine-disassembled spindles. The results strongly suggest that demecolcine induces ooplasmic protrusion by destroying the oocyte spindle and activating MPF and MAPK. Studies in somatic cells also indicated that treatment with microtubule-interacting agents increased MPF activities [23]–[25]. In this study, while 95% of the demecolcine-treated oocytes were MAD2-postive and injection of anti-MAD2 antibodies inhibited ooplasmic protrusion and activation of MPF and MAPK, all of the oocytes with MG132-induced protrusions were MAD2-negative and injection of anti-MAD2 antibodies had no effect. It was shown that defects in spindle assembly or spindle-kinetochore attachment, or artificial depolymerization of microtubules, activated the mitotic checkpoint, which arrested cells prior to the metaphase-anaphase transition with unsegregated chromosomes, stable cyclin B and elevated MPF activity [26], [27]. Further studies confirmed that MAD2 activated MPF mainly by preventing cyclin B proteolysis [23], [24], [28], [29].

Although studies suggest activation of MAPK family members by microtubule inhibitors in somatic cells [30]–[32], it has rarely been studied in the oocyte [22], [33]. Furthermore, the mechanism by which microtubule dysfunction activates MAPK is unknown. In this study, while treatments that activated both MPF and MAPK induced ooplasmic protrusion, treatments that activated MPF while inhibited MAPK activation did not (Table 1). In both freshly matured and aged oocytes without protrusions after demecolcine treatment, while MPF activities increased, MAPK activity did not. MG132 and caffeine which are known to specifically activate MPF increased activities of both MPF and MAPK. Furthermore, while treatment with ROS (specific CDK1 inhibitor) inhibited activation of MAPK as well, treatment with U0126 (specific inhibitor of MEK1/2) inhibited only MAPK. While some studies showed that MPF triggered activation of MAPK [34]–[36], others demonstrated a converse situation [37]–[40]. Studies in rodent [41]–[43] and goat [44] oocytes suggest that MPF might be an upstream regulator of the MAPK/Mos pathway during meiotic maturation. Furthermore, the possible role of MAPK in MPF activation in mammalian oocytes was ruled out by the observation that oocytes derived from mos-knockout mice, which are unable to activate MAPK, display a normal pattern of MPF activation during germinal vesicle breakdown and Pb1 emission [45]. After Pb1 emission, however, MPF cannot be properly reactivated in some of the c-mos knockout oocytes. Thus, the present results suggested that (1) microtubule dysfunction activated MAPK by activating MPF and (2) MPF activated MAPK during actomyosin assembly at the metaphase II (MII) stage of oocytes.

**Table 1:** Interactions between MPF, MAPK and RhoA on enrichment of actin and myosin during ooplasmic protrusion following different treatments of goat oocytes.

Treatments	Protrusion	MPF	RhoA	MAPK	Actin enrichment	Myosin enrichment	Spindle integrity
D	+	+	+	+	+	+	−
D−	−	+	−	−	−	−	−
D+U	−	+	+	−	−	−	−
D+Y	−	+	−	−	−	−	−
D+R	−	−	−	−	−	−	−
M	+	+	NA	+	+	+	+
M+U	−	+	NA	−	−	−	+
M+Y	−	+	−	−	−	−	+
M+Y+OA	−	+	−	+	+	−	+
M+R	−	−	NA	−	−	−	+
OA.5h	−	−	−	+	+	−	+
OA4h	+	+	+	+	+	+	+
OA4h+R	−	−	−	+	+	−	+
OA4h+U	−	−	NA	−	−	−	+

Abbreviations: D: 0.8 ng/ml demecolcine; D-: Oocytes without protrusion after D; U: 20-µM U0126; Y: 400-µM Y27632; R: 400-µM ROS; M: 5-µM MG132; C: 1-mM caffeine; OA.5h: 0.5-µM OA for 0.5 h; OA4h: 0.1-µM OA for 4 h; +: In the presence of; NA: Not assayed. All treatments were performed for 30 min unless otherwise specified.

Among the drugs used in this study, while demecolcine prevented proteolysis of cyclin B by activating spindle assembly checkpoints, MG132, the specific inhibitor of proteasome catalytic activity, prevented cyclin B degradation directly [46], [47]. Caffeine increased MPF activity by inhibiting Myt1/Wee1 and dephosphorylating Cdc2 [48], [49]. Thus, the MPF in MII oocytes may be activated either by cyclin B accumulation or by Cdc2 dephosphorylation. While treatment with OA for 0.5 h increased only MAPK activity, OA treatment for 4 h increased MPF activity as well in this study. Several studies indicated that OA activated MPF as well as MAPK [41], [50], [51]. OA may activate MPF either directly by inhibiting protein phosphatase 1 and 2A that would otherwise inactivate Cdc25 [52] or indirectly by activating MAPK which activates p90(rsk) and down-regulates Myt1 [39], [40]. In this study, when oocytes were treated with OA for 4 h, neither MPF nor MAPK activities increased in the presence of U0126; however, while MPF activation was inhibited, MAPK activity increased as usual in the presence of ROS. This suggested that OA activated MPF by activating MAPK, a situation similar to that of c-mos knockout mouse oocytes in which MPF could not be properly activated after first meiosis [45]. Together, the results suggest that in MII oocytes, MPF and MAPK can each activate the other depending on circumstances.

However, the mechanism by which MPF activates MAPK has not been reported. In this study, when oocytes were treated with demecolcine, MG132 or caffeine in the presence of the ROCK inhibitor Y27632, protrusion and MAPK activation was blocked while the MPF activity increased as usual (Table 1). Furthermore, RhoA-GTP assay confirmed that the elevated MPF activities activated RhoA which then activated MAPK during ooplasmic protrusion. In keeping with the present results, studies using somatic cells indicated that microtubule minimization or depolymerization activated RhoA [2], [3], and that Rho GTPase activated components in the MAPK signaling pathway [53]–[56]. In Xenopus oocytes, Rho-associated protein kinase alpha potentiated insulin-induced MAPK activation [57]. However, Rho activation by increased MPF activities has not been reported, but on the contrary, a decrease in MPF activities was found to activate RhoA by activating the guanine nucleotide exchange factor (GEFs) in both somatic cells [58], [59] and mouse oocytes [60].

Since Elbaz et al. [60] also observed that many of the ECT2 (GEFs) depleted oocytes that failed to extrude Pb1 displayed an elongated protrusion reminiscent of the induced ooplasmic protrusion of this study, we postulated that the recruitment of actin and myosin into contractile rings and the contraction of the ring might be two separate steps involving different regulation mechanisms; while the contraction requires activated ECT2 for RhoA activation, assembly of the ring might be regulated by a different RhoA-activating mechanism. Recent studies in yeast have shown that CDK/cyclin complex promotes actin polarization for hyphal growth by inhibitory phosphorylation of GTPase activating proteins (GAPs) that would otherwise inactivate Rho [61], [62]. In this study, when goat oocytes with demecolcine-induced protrusions were chemically activated with ionomycin and CHX, MPF decreased while RhoA-GTP increased sharply, and the actomyosin ring contracted and pinched off as Pb2 with the whole complement of chromosomes. Y27632 inhibited extrusion of Pb2 but had effects on neither the MPF nor MAPK dynamics. This suggested that (1) RhoA activation was essential for the constriction of actomyosin rings after oocyte activation, and (2) while increased MPF activities activated RhoA possibly by inactivating GAPs during the assembly, decreased MPF activities activated RhoA probably by activating GEFs during the constriction of the actomyosin ring. During mouse oocyte maturation, coincident with the cortical localization of the meiotic spindle was the formation of a microvillus- and cortical granule-free area and a thickening of the actin layer in this region of the egg cortex [10], and it was found that most of the mouse oocytes completed formation of the cortical granule-free domain at the metaphase I stage [63]. In fission yeast, while formation of the contractile ring was completed at 30 min, contraction of the ring was not started until 37 min after the onset of spindle pole body separation [64].

In this study, oocytes with protrusions were always characterized by enrichment of both actin and myosin in the protruding domains, and treatment with either actin or myosin inhibitor completely inhibited ooplasmic protrusion. Requirements for actin-myosin interactions have been reported during cytokinesis of various cell types [7], [65], [66], but the signaling events involved have not been fully elucidated. Studies using somatic cells suggest that (1) MAPK activities are involved in the regulation of actin reorganization [67]–[70]; (2) MAPK enhances myosin light chain kinase (MLCK) activity leading to activation of myosin light chains (MLC) [71]–[73], which is important for recruitment of myosin into the contractile ring [74]; (3) MPF activates MLC [75], [76] either by inhibiting myosin phosphatase-targeting subunit (MYPT) [77] which would otherwise inactivate MLCK [78]–[80], or by phosphorylating caldesmon which would otherwise inactivate actomyosin ATPase [4], [81]; and (4) ROCK activates myosin II by inactivating MYPT [3]. However, interactions between MPF, MAPK and Rho GTPase in this context have not been reported. This study showed that while actin enrichment required only the increase in MAPK activity during ooplasmic protrusion, myosin enrichment required elevated activities of MPF as well as MAPK and RhoA GTPase (Table 1), suggesting that while MAPK controls actin recruitment on its own, MPF promotes myosin recruitment in a RhoA- and MAPK-dependent manner during actomyosin assembly. Since both MPF and ROCK inactivated MYPT in somatic cells and that MPF activated RhoA in this study, our hypothesis to explain the dependence of MPF on MAPK and ROCK for myosin recruitment is that (1) MPF inactivates MYPT via RhoA and (2) only when MYPT is inactivated can the elevated MAPK activity activate MLCK and thereby myosin II.

In this study, injection of mouse sperm head into intact goat oocytes induced a second ooplasmic protrusion after treatment with demecolcine or MG132. However, no protrusion formed when enucleated oocytes were treated with MG132 although both MPF and MAPK activities increased significantly. Thus, ooplasmic protrusion requires an interaction between the elevated MPF/MAPK activities and the chromatin, which can be from a different species. The chromosome spindle was found to determine the localization of the actin-rich but cortical granule-free domain during polarization of maturing mouse oocytes [10]–[12], [15], [16], [82]. When the chromosomes became scattered following microtubule disruption, however, a cortical granule-free domain was observed over each individual chromosome [11], [83]. Furthermore, mouse sperm chromatin, even DNA-coated beads, could induce polarization of mouse oocytes [12], [15], [16], [84] after microinjection. According to Deng and Li [84], the signals that emanate from the sperm chromosomes to induce spindle formation and cortical reorganization are qualitatively and quantitatively different; while injection of loosely compact chromatin from round spermatids induced both cortical reorganization and spindle formation, leading to polar body extrusion, injection of highly compact chromatin from mature spermatozoa only allowed cortical reorganization without spindle formation and polar body extrusion. Thus, the results show a similarity between the chemical-induced cortical protrusion and polarization of oocytes.

**Figure 6:**
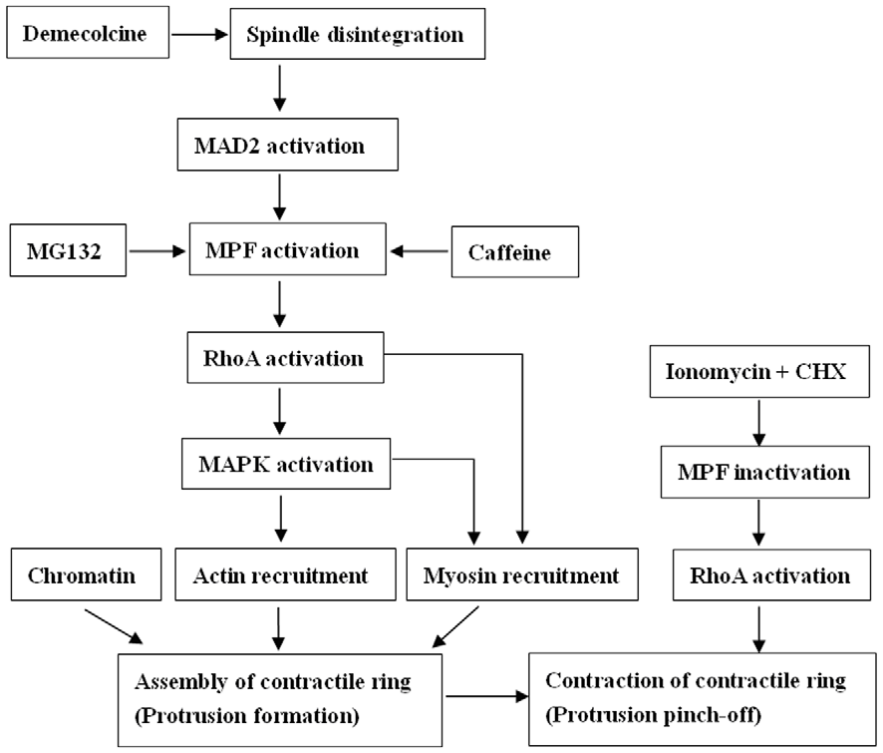
The signaling pathways leading to the assembly and contraction of actomyosin rings during chemical-induced oocyte cytokinesis. Refer to the text for detailed explanations.

To summarize the major findings of this study (Figure 6), demecolcine activates MPF by disassembling spindle microtubules and activating MAD2 while MG132 and caffeine activate MPF directly. The increased MPF activity activates RhoA possibly by inactivating GAPs. The activated RhoA activates MAPK. While the elevated MAPK activity recruits actin on its own, the increased MPF activity recruits myosin in a RhoA- and MAPK-dependent manner. From data obtained in other systems, it is possible that RhoA inactivates MYPT, which allows the activation of MLCK by MAPK leading to the recruitment of myosin into the contractile ring. Chromatin or DNA is essential for the assembly of actomyosin rings. When oocytes with demecolcine-induced protrusions were treated with ionomycin and CHX, the MPF activity decreases, activating RhoA possibly by activating GEFs. The active RhoA induces contraction of the ring resulting in cytokinetic abscission of the protrusion (Pb2 extrusion).

## Materials and Methods

All chemicals and reagents were purchased from Sigma Chemical Company (St. Louis, MO, USA) unless otherwise specified. Oocyte culture was carried out at 38.5°C in 5% CO_2_ in humidified air, unless otherwise specified.

### Collection and *in vitro* maturation (IVM) of oocytes

Oocytes collected from goat ovaries [22] were cultured for maturation in droplets of TCM-199 (Gibco, Grand Island, New York, USA) supplemented with 10% FCS (Gibco), 1 µg/ml 17 β-estradiol, 24.2 mg/L sodium pyruvate, 0.05 IU/ml FSH, 0.05 IU/ml LH and 10 ng/ml EGF. At 19 h of culture, oocytes were freed of cumulus cells and those with a first polar body (Pb1) but without spontaneous ooplasmic protrusions were selected for further treatments.

### Treatment of oocytes to regulate the assembly and contraction of contractile rings

To induce or inhibit ooplasmic protrusion, oocytes were cultured in Dulbecco's phosphate-buffered saline (D-PBS) supplemented with different drugs for 30 min. At the end of treatment, oocytes were examined under a stereomicroscope and were divided into groups with or without ooplasmic protrusions for further treatments.

To induce contraction of the actomyosin ring, oocytes with demecolcine-induced ooplasmic protrusions and the control oocytes collected at 19 h of IVM were first exposed to 5-µM ionomycin for 5 min at room temperature, and then cultured in the CR1aa medium containing 10-µg/ml cycloheximide (CHX) with or without 400-µM Y27632. Oocytes were observed under a phase contrast microscope for extrusion of Pb2 or assayed for MPF/MAPK and RhoA activities at 0.5, 1 or 2 h of culture. Micromanipulators were used to move the Pb2 to confirm cytokinetic abscission.

Roscovitine (ROS, 20 mM), U0126 (10 mM), okadaic acid (OA, 100 µM), MG132 (5 mM), Y27632 (20 mM), cytochalasin B (CB, 5 mg/ml), blebbistatin (34.2 mM) and ionomycin were dissolved in dimethyl sulfoxide (DMSO), while demecolcine (10 µg/ml) was dissolved in Hank's balanced salt solution (HBSS), caffeine (100 mM) was dissolved in D-PBS and CHX in water. All the stock solutions were stored in aliquots at −20°C except for demecolcine which was stored at 4°C until use.

### Immunofluorescence microscopy

All the procedures were conducted at room temperature unless otherwise specified. Oocytes were washed 3 times in D-PBS between treatments. Oocytes were (1) freed of zona pellucida by treatment with 0.5% pronase (Roche Diagnostics GmbH, Mannheim, Germany) in D-PBS at 37 °C for 3–5 min; (2) fixed with 4% paraformaldehyde in the PHEM buffer (60 mM Pipes, 25 mM Hepes, 10 mM EGTA and 4 mM MgSO_4_, pH 7.0) for at least 30 min; (3) treated for 10–15 min in 1% Triton-X 100 in PHEM; (4) blocked in PHEM containing 1% BSA and 100 mM glycine at 4 °C overnight.

#### Triple staining of α-tubulin, actin and chromatin

Blocked oocytes were incubated (1) for 1 h in PHEM containing fluorescein isothiocyanate (FITC)-conjugated anti-α-tubulin monoclonal antibodies (1∶50) to stain tubulin; (2) for 1 h in PHEM containing tetramethylrhodamine B isothiocynate (TRITC)-conjugated phalloidin (1∶200) to label actin; (3) for 15 min in D-PBS with 10 µg/ml Hoechst 33342 to stain chromosomes.

#### Double staining of MAD2 and chromatin

Blocked oocytes were incubated (1) overnight in rabbit anti-MAD2 antibody (1∶50, Santa Cruz, Biotechnology, Inc.) in 1% BSA/PHEM with 100 mM glycine at 4°C; (2) for 1 h with FITC-conjugated goat-anti-rabbit IgG (1∶200, Santa Cruz, Biotechnology, Inc.) in 1% BSA/PHEM with 100 mM glycine; (3) for 15 min in D-PBS with 10-µg/ml propidium iodide to stain chromosomes.

#### Triple staining of myosin IIA, actin and chromatin

Blocked oocytes were incubated (1) for 2 h in rabbit anti-myosin IIA antibody (1∶50, Santa Cruz, Biotechnology, Inc.) in 1% BSA/PHEM with 100 mM glycine; (2) for 1 h with FITC-conjugated goat-anti-rabbit IgG (1∶200) in 1% BSA/PHEM with 100 mM glycine; (3) for 1 h to stain actin as described above; (4) for 15 min in D-PBS with 10 µg/ml Hoechst 33342 to stain chromosomes.

#### Laser confocal microscopy

The stained oocytes were mounted on glass slides and observed with a Leica laser scanning confocal microscope. Hoechst 33342 labeled chromatin was excited with the 405 nm line of a diode laser. The FITC/PI and TRITC fluorescence was obtained by excitation with 488 and 533 nm lines of an Ar/ArHr laser and the emitted light was passed through 488 and 533 nm filters, respectively. The individual optical sections were pseudo-colored and digitally recombined when necessary into a single composite image using the Leica Confocal Software.

### Histone H1 and MAP kinase assay

Ten oocytes from each treatment were washed 3 times in the kinase buffer (15-mM 3-[n-morpholino] propanesulfonic acid [MOPS], pH 7.2, containing 80-mM glycerophosphate, 10-mM EGTA, 15-mM MgCl_2_, 0.1-mM PMSF, 10 µg/ml leupeptin, 10 µg/ml aprotinin, and 10 µg/ml cAMP-dependent protein kinase inhibitor peptide), transferred to10-µl kinase buffer contained in a 1.5 ml microfuge tube and stored frozen at −70°C. The frozen samples were subjected to 4 to 5 times freezing and thawing to prepare lysates. Kinase reactions were initiated by the addition of 10-µ1 substrate buffer containing 2 mg/ml histone H1 or 1 mg/ml myelin basic protein (MPB), 2-mM dithiothreitol (DTT) and 20-µCi/ml [γ-^32^P] ATP to each sample. The reactions were carried out for 50 min at 36°C and terminated by the addition of an equal volume of double-strength SDS sample buffer containing β-mercaptoethanol. Kinase reaction products were boiled for 3 min and separated by 12% linear gradient SDS-PAGE. Gels were exposed to phosphor-screens. Data acquisition was the actual scanning of sample images with the Cyclone® Plus Storage Phosphor System to create an image file that can be analyzed by the OptiQuant™ Image Analysis Software. The kinase activity values of newly matured goat oocytes collected 19 h of IVM were arbitrarily set as 100%, and the other values were expressed relative to this activity. The amount of kinase reaction product used for SDS-PAGE was strictly controlled (20 µl) for each sample, and three samples were analyzed for each treatment.

### Microinjection of anti-MAD2 antibodies into goat oocytes

Microinjection of anti-MAD2 antibodies was performed in D-PBS using a Leica inverted microscope equipped with two micromanipulators. A goat oocyte was held to the holding pipette at the 9 o'clock position and then rotated until the oocyte side with Pb1 was around the 12 o'clock position. The injection pipette (4-µm in inner diameter with a beveled fine point) containing antibodies was positioned at the 3 o'clock position and advanced to penetrate the zona pellucida and the oolemma. Once inside the oolemma, a volume of about 30-pl of the antibodies (0.2 mg/ml in D-PBS) was injected into the oocyte. Control oocytes were injected with the same volume of goat IgG (0.5 mg/ml in D-PBS) or D-PBS. After injection, oocytes were cultured for 30 min in maturation medium before treatments to induce ooplasmic protrusions.

### Rho-GTPase activity assay

From each treatment, 150 or 50 oocytes were collected for RhoA-GTP or total RhoA analysis, respectively. Oocytes were (1) washed 3 times in a sample buffer containing 50-mM Tris pH 7.2, 1% Triton X-100, 0.5% sodium deoxycholate, 0.1% SDS, 60-mM n-octyl glucopyranoside, 500-mM NaCl, 10-mM MgCl_2_, 10 µg/ml leupeptin, 10 µg/ml aprotinin and 1-mM phenylmethylsulphonyl fluoride (PMSF); (2) transferred to a 0.2-ml microfuge tube with 20-µl buffer and lysated by 3–5 times freezing and thawing; (3) lysates were incubated with 5-µg of a glutathione-agarose bound GST-tagged rhotekin Rho binding domain (Upstate Biotechnology in USA) at 4°C for 45 min with gentle rocking; (4) beads were collected by centrifugation at 15,000 rpm for 30s at 4°C and washed twice in the sample buffer; (5) 15-µl sample buffer containing beads in each tube were frozen at −70°C; (6) frozen beads were resuspended in 15-µl of 2×Laemmli sample buffer, boiled for 5 min and separated by 12% linear gradient SDS-PAGE; (7) the protein samples were transferred onto PVDF membranes in transferring buffer containing 0.1M Tris, 0.192M glycin and 5% methanol in TBST (TBS with 0.1% Tween 20) at 2mA/cm^2^ for 30 min with AE-6675 HorizBlot (ATTO corporation in Japan); (8) membranes were blocked for 1 h with TBST containing 3% BSA and incubated with (a) anti-RhoA antibody (1∶500 dilution, Abcam in UK) in TBST with 3% BSA at 4°C overnight, (b) horseradish peroxidase-conjugated antibody (1∶1000 dilution, Sizheng Biotechnology, China) in TBST/3% BSA for 1 h and (c) Immobilon Western reagent (Millipore Corporation in USA); (9) membranes were exposed to x-ray film. The relative quantity of RhoA-GTP was determined with Image-Pro Plus Software by analyzing the sum density of each protein band image. The quantity values of newly matured oocytes collected 19 h of IVM were arbitrarily set as 100%, and the other values were expressed relative to this activity. β-actin in each sample was also analyzed as control using the anti-β-actin antibody. Total lysate was immunoblotted with the same RhoA antibody to illustrate equality in oocyte RhoA across conditions.

### Microinjection of mouse sperm heads or tails into goat oocytes

Masses of dense sperm were collected from mouse caudae epididymides [85] and placed in a test tube containing 500-µl D-PBS. After mixing, the resulting sperm suspension was sonicated for 5 min to separate sperm heads from the tails. The suspension was then heated in a water bath at 60–65°C for 40 min and 1∶1 diluted in D-PBS before microinjection. The Pb1 and 1/5–1/4 of the ooplasm underneath were removed from some oocytes using an enucleation pipette (25 µm in inner diameter). To inject sperm heads or tails, an oocyte was held to the holding pipette at the 9 o'clock position and then rotated until the oocyte side with Pb1 was around the 12 o'clock position. The injection pipette (8–10 µm in inner diameter) containing a sperm head or a tail was advanced to penetrate the zona and the oolemma at the 3 o'clock position. Care was taken to place sperm heads or tails into the egg cortex as close to the plasma membrane as possible. After microinjection, the oocytes were cultured for 30 min in maturation medium before treatment for protrusion induction.

### Data analysis

There were at least three replicates for each treatment. Percentage data were arc sine transformed and analyzed with ANOVA; a Duncan multiple comparison test was conducted to locate differences. The soft ware used was Statistics Package for Social Science (SPSS 11.5, Chicago, IL, USA). Data were expressed as mean ± SE and P<0.05 was considered significant.
